# Accumbal Adenosine A_2A_ Receptors Enhance Cognitive Flexibility by Facilitating Strategy Shifting

**DOI:** 10.3389/fncel.2019.00130

**Published:** 2019-04-11

**Authors:** Jianhong Zhou, Beibei Wu, Xiangxiang Lin, Yuwei Dai, Tingting Li, Wu Zheng, Wei Guo, Sergii Vakal, Xingjun Chen, Jiang-Fan Chen

**Affiliations:** ^1^Molecular Neuropharmacology Laboratory, School of Optometry and Ophthalmology and Eye Hospital, Wenzhou Medical University, Wenzhou, China; ^2^State Key Laboratory of Optometry & Vision Science, Wenzhou, China

**Keywords:** adenosine A_2A_ receptors, nucleus accumbens, dorsomedial striatum, attentional set-shifting, reversal learning, motivation, attention

## Abstract

The deficits of cognitive flexibility (including attentional set-shifting and reversal learning) concomitant with dysfunction of the striatum are observed in several neuropsychiatric disorders. Rodent and human studies have identified the striatum [particularly the dorsomedial striatum (DMS) and nucleus accumbens (NAc)] as the critical locus for control of cognitive flexibility, but the effective neuromodulator and pharmacological control of cognitive flexibility remains to be determined. The adenosine A_2A_ receptors (A_2A_Rs) are highly enriched in the striatopallidal neurons where they integrate dopamine and glutamate signals to modulate several cognitive behaviors, but their contribution to cognitive flexibility control is unclear. In this study, by coupling an automated operant cognitive flexibility task with striatal subregional knockdown (KD) of the A_2A_R *via* the Cre-loxP strategy, we demonstrated that NAc A_2A_R KD improved cognitive flexibility with enhanced attentional set-shifting and reversal learning by decreasing regressive and perseverative errors, respectively. This facilitation was not attributed to mnemonic process or motor activity as NAc A_2A_R KD did not affect the visual discrimination, lever-pressing acquisition, and locomotor activity, but was associated with increased attention and motivation as evident by the progressive ratio test (PRT). In contrast to NAc A_2A_Rs, DMS A_2A_Rs KD neither affected visual discrimination nor improved set-shifting nor reversal learning, but promoted the effort-related motivation. Thus, NAc and DMS A_2A_Rs exert dissociable controls of cognitive flexibility with NAc A_2A_Rs KD selectively enhancing cognitive flexibility by facilitating strategy shifting with increased motivation/attention.

## Introduction

Cognitive flexibility is an essential executive function that enables individuals and species to adapt to new surroundings in the constantly changing environment and can be divided into two distinct components including attentional set-shifting (extra-dimensional shifting) and reversal learning (intra-dimensional shifting). The impairment of cognitive flexibility is often observed in several mental disorders concomitant with dysfunction of the basal ganglia, including attentional deficit and hyperactivity disorder (ADHD; Reeve and Schandler, 2001), early Parkinson’s disease (Cools et al., 2001), schizophrenia (Pantelis et al., 1999), drug addiction (Kalivas and Volkow, 2005) and autism (Leung and Zakzanis, 2014). The effective pharmacological strategies to improve the deficit in cognitive flexibility in neuropsychiatric disorders are critically needed.

Rodent and primate studies have revealed the distinct cortical-subcortical circuits subserved cognitive flexibility (Birrell and Brown, 2000; McAlonan and Brown, 2003; Ragozzino, 2007). Furthermore, as the primary brain region receiving cortical glutamatergic inputs, striatum also plays an essential role in neuronal control of cognitive flexibility. The striatum is an anatomically and functionally heterogeneous structure that can be distinguished into the dorsomedial striatum (DMS, involving goal-directed behavior), dorsolateral striatum (involving habit formation) and the ventral striatum [nucleus accumbens (NAc), involving reward, motivation and emotion; Yin and Knowlton, 2006; Bagot et al., 2015; Li et al., 2016]. The dorsal striatum receives glutamatergic excitatory afferents from the sensorimotor, prefrontal cortical areas and the intralaminar thalamic nuclei (Hunnicutt et al., 2016; Kato et al., 2018), as well as dopaminergic innervations from the substantia nigra pars compacta (Horvitz, 2002). The ventral striatum mainly receives convergent glutamatergic inputs/projections from the ventral hippocampus (vHIP), medial prefrontal cortex (mPFC), basolateral amygdala (BLA) and paraventricular thalamus (French and Totterdell, 2002; Sesack and Grace, 2010; Britt et al., 2012), and dopaminergic inputs from the ventral tegmental area (VTA; Goto and Grace, 2008). Accordingly, NAc has been shown to be critical to control cognitive flexibility (Haluk and Floresco, 2009; Ding et al., 2014; Cui et al., 2018). This NAc control of cognitive flexibility may be associated with NAc ability to modulate attention (Christakou et al., 2004; Salgado and Kaplitt, 2015), working memory (Takahashi et al., 2011; Laplante et al., 2012) and goal-directed behavior (Mannella et al., 2013). Similarly, DMS has also an important role in the control of cognitive flexibility and other cognitive behavior (Li et al., 2016, 2018; Kato et al., 2018; Zhu et al., 2018).

Both glutamatergic and dopaminergic signaling in the striatum are critical for the control of cognitive flexibility by strategy shifting. For example, the glutamatergic signaling from the mPFC is engaged, specifically, in mediating attentional set-shifting extradimensionally, while the glutamate signaling from the orbitofrontal cortex (OFC) selectively controls reversal learning intradimensionally (Birrell and Brown, 2000; Ragozzino, 2007). Moreover, the dopaminergic signaling regulates some elements of behavior flexibility as well as various learning and memory-associated behaviors (Haluk and Floresco, 2009; Cui et al., 2018). In addition, other neuromodulators such as endocannabinoid (Varvel and Lichtman, 2002; Klugmann et al., 2011), acetylcholine (Aoki et al., 2015; Prado et al., 2017), GABA (Yawata et al., 2012) and BDNF (Parikh et al., 2016a,b) and adenosine (Wei et al., 2011) have been implicated in the control of cognitive flexibility. However, the control of cognitive flexibility by neuromodulators other than glutamate and dopamine systems in the different striatal regions is still largely unexplored.

The adenosine A_2A_ receptors (A_2A_Rs) are highly enriched in the striatopallidal neurons (Svenningsson et al., 1999) where A_2A_Rs interact with dopamine D_2_ receptors (Schiffmann et al., 2010) and N-methyl-D-aspartate receptors (NMDARs; Higley and Sabatini, 2010), as well as metabotropic glutamate 5 receptors (mGlu5; Ferré et al., 2002). Striatopallidal A_2A_Rs can integrate glutamatergic and dopaminergic signals to control striatal synaptic plasticity and various cognitive behaviors in both normal and abnormal conditions (Chen et al., 2013; Chen, 2014). Recent studies from our and other labs have demonstrated that activation of the striatopallidal A_2A_Rs exerts inhibitory control of various cognitive behaviors such as working memory and goal-directed behavior (Wei et al., 2014; Li et al., 2016, 2018). Thus, we propose that the A_2A_R inaction represents a novel target for reversing cognitive deficit in neuropsychiatric disorders (Chen, 2014). This proposal has high translational potential given that the A_2A_R antagonist is in clinical phase III trial for the treatment of Parkinson’s disease with a notable safety profile (Chen et al., 2013). However, the exact role of striatal A_2A_Rs in the control of cognitive flexibility (i.e., attentional set-shifting and reversal learning) is mostly unclear. Limited studies showed that A_2A_R inactivation is associated either with impaired (Amodeo et al., 2018) or enhanced (Wei et al., 2011) or no effect (O’Neill and Brown, 2007) on cognitive flexibility. Moreover, as the dorsomedial and dorsolateral striatum A_2A_Rs exert distinct control of goal-directed and habitual behaviors, respectively (Li et al., 2016), the specific contributions of the striatopallidal A_2A_Rs in NAc and DMS to the control of strategy shifting remain to be determined.

In this study, we adapted the automated operant cognitive flexibility task which minimizes the procedural disadvantages and vulnerability to manual error and subjective interpretation of the cross-maze task and the digging task to test behavioral flexibility in rodents (Haluk and Floresco, 2009; Brady and Floresco, 2015; Parikh et al., 2016a). This task (including visual discrimination, attentional set-shifting, and reversal learning) placed heavier emphasis on response conflicts and shared similar features to the Wisconsin Card Sorting Task established to assess the cognitive flexibility of human beings (Monchi et al., 2001). By coupling this cognitive flexibility task with the Cre-loxP-mediated focal knockdown (KD) of A_2A_Rs in the DMS and NAc, we critically determined the effects of DMS and NAc A_2A_R on cognitive flexibility. We further explored the possible role of a motivational factor in the modulation of A_2A_R control of strategy shifting by progressive ratio test (PRT).

## Materials and Methods

### Subjects

The animal protocols were approved by the Institutional Ethics Committee for Animal Use in Research and Education at Wenzhou Medical University, China. All mice were housed at a constant temperature (24 ± 0.5^o^C) with a relative humidity of 60 ± 2% and controlled by a 12-h light-dark cycle (light on at 8:00 A.M.). Except for the periods of food-restriction for the purpose of behavioral training and testing, all mice were given *ad libitum* access to food and water. The A_2A_R^flox/flox^ mice were generated and then backcrossed to C57BL/6 for 10 generations to generate congenic A_2A_R^flox/flox^ in the C57BL/6 genetic background, and characterized as we described previously (Shen et al., 2008; Augusto et al., 2013).

### The Cre-loxP-Mediated Conditional A_2A_Rs Knockdown Strategy

Male A_2A_R^flox/flox^ mice, aged 8–12 weeks, were used in the experiments. Conditional KD of the A_2A_R gene was achieved by injecting Cre recombinase-expressing AAV to the DMS (AP, +0.98 mm; ML, ±1.20 mm; DV, 2.50 mm) or NAc (AP, +1.3 mm; ML, ±1.00 mm; DV, 3.90 mm). Specifically, AAV8-CAG-Cre-ZsGreen (200 nl) was injected bilaterally into A_2A_Rs^flox/flox^ mice *via* a Hamilton injection syringe to achieve focal KD of A_2A_Rs in targeted subregions. A_2A_R^flox/flox^ mice injected with AAV8-CAG-ZsGreen were used as the control. The mice were allowed to recover for 3 weeks, and the conditional KD of A_2A_Rs was carried out before behavioral training.

### Open-Field Test and Spontaneous Alternation Test in the Y-maze

For the open-field test, mice were placed in the center of a white, dimly lit open-field chamber (40 × 40 cm) and allowed to explore the environment for a total of 10 min freely. The center of the open-field was defined as >20 cm apart from all four walls. Total movement distance and the time spent in the center and periphery were recorded by an automated video tracking system (EthoVision system, Noldus). For spontaneous alternation test in the Y-maze, all the mice were placed into a Y-maze and allowed to navigate for 8 min freely. The sequence of animal entries to each arm and the number of entries were recorded. Correct spontaneous alternation was defined as the continuous entry into three arms (such as 1, 2, 3 or 1, 3, 2) as described previously (Zheng et al., 2018).

### Mouse Operant Cognitive Flexibility Task

We adapted standard operant conditioning chambers (MED Associates., Albans, VT, USA) for an automated operant cognitive flexibility task as described previously with slight modifications (Haluk and Floresco, 2009; Brady and Floresco, 2015; Parikh et al., 2016a). All the operant procedures and data collection in this task can be automatically controlled by a customized program. Briefly, in the operant conditioning chambers, two retractable levers were mounted at either side of the receptacle with a central reward port attached to a fluid dipper between them, and a light stimulus was placed above each lever. Animals were manually handled, and their body weight was restricted to 80%–85% of their original weight before the beginning of the test.

#### Autoshaping and Side Preference Task

When shaping in the operant chambers, all the mice had 1 day magazine training in which as long as the mice poked the central reward port, they would receive 10 μl of 20% sucrose solution as a reward. After that, all the mice were autoshaped on an FR-1 schedule of reinforcement in which mice were required to press the lever to get the reward (each lever press leading to one reinforcement delivered). In this training, only one lever was present, but the reinforced lever (left or right lever) was counterbalanced across animals and training days to prevent the mice from forming a lever bias. After meeting the criterion of getting 50 rewards per session for two consecutive days, mice were advanced to the retractable lever training sessions to familiarize them with the extension and retraction of the levers. In these training sessions, each trial consisted of a lever presentation (either left or right) for 8 s, and the lever was extended in a pseudorandom order with no more than two consecutive trials extending the same lever. Each lever press response was rewarded and terminated the lever extension. If the animal did not respond within 8 s, the lever would automatically be retracted, and the trial was recorded as an omission. To control for any novelty effect that might be associated with the visual stimulus during the subsequent stage of the task, the activated lever was randomly associated with an unpredictably occurring illumination of the panel light. Trials were presented with an inter-trial interval (ITI) of 9 ± 3 s. The day after reaching the criteria (40 rewards and ≤20% omissions for two consecutive days), the side preference of animals was assessed. The side preference task consisted of 10 trials. In every trial, both levers were inserted into the chamber simultaneously, and the initial reward was available after responding on either lever, but the mice had to respond on the lever opposite to the one chosen initially to get a reward upon the following response. If the mice pressed the same lever as the initial choice, no reinforcement would be delivered. This task continued until the animals chose the lever opposite to that chosen initially and the number of responses on each lever would be recorded. After choosing both levers with an ITI of 12 s, a new trial commenced. The lever (right or left) that mice responded on the initial choice of a trial was recorded and counted as its bias lever. If the total number of responses on each lever was comparable, the lever that mice chose initially six or more times over 10 total trials was considered its side bias. However, if a disproportional number of responses was made on one lever (greater than a 2:1 ratio), the lever was considered its side bias.

After the side preference testing, all the mice were officially progressed to the mouse operant cognitive flexibility task, which consisted of three different phases: visual discrimination, strategy set-shifting, and reversal learning.

#### Visual Discrimination Phase

During the visual discrimination phase, the two levers were present at the same time, and either of the levers was randomly illuminated with the light stimulus, and mice were required to discern the lever with an activated cue light to get a reward within 8-s test period after the lever extension. All trials were started with a 2-s acoustic stimulus, the lever and cue light were automatically retracted and turned off if any lever pressing happened or no response happened within 8 s (the trial counted as an omission response). The ITI was 9 ± 3 s. A lever press response on the cued lever was scored as “correct response,” whereas pressing the non-illuminated lever was defined as “incorrect response.” Each session included 40 trials, and all the mice were trained for one session per day. When the animals were able to meet the criterion with 75% correct responses for three consecutive days in the phase of visual discrimination, all the mice were advanced to the phase of the attentional set-shifting.

#### Set-Shifting Phase

During this phase, animals were required to shift to the lever-pressing response task, which reinforced animals for responding on the lever opposite their side preference, regardless of stimulus light (cue) illumination. The experimental parameters remained identical to the visual discrimination phase except that the contingencies were altered in such a way that the animals were requested to press the lever other than their bias lever to get reward irrespective of the cue presentation which remained pseudorandom. For example, if the mice bias lever is the left lever, in this phase the mice have to press the right lever to get a reward and ignore the cue presentation. Animals that had successfully attained the criterion (80% correct responses for three consecutive days) at this stage were moved to the reversal learning phase. The visual discrimination task was the “Set” task in this phase, and the response task was the “Shift” task in this phase. The Set-shifting phase also can be termed as extradimensional shifting which referred to the ability to actively suppress a previously learned response strategy while acquiring a new competing strategy, particularly across stimulus dimensions—for example, switching from performing visually-based discrimination to lever-pressing response discrimination in our behavioral paradigm.

#### Reversal Phase

During this phase of training, the reinforced lever was reversed again; animals were required to press the opposite lever, which was assigned to the correct lever during the preceding phase (set-shifting) regardless of the position of the illuminated cue until reaching the criterion (80% correct responses for three consecutive days). For example, the mice have to press the left lever to get a reward in this phase, if the mice were required to press the right lever to get a reward in the Set-shifting phase. Reversal learning also can be termed as intradimensional shifting which involved a change in response strategy but within the same stimulus dimension—for example, switching from a left lever-based reinforcement to a right lever-based reinforcement in our reversal phase.

The number of correct responses, errors, omissions and response latencies were automatically obtained for each behavioral session. Response accuracies were calculated for each session according to the formula: correct responses/(correct + incorrect responses) × 100%. The total number of performed trials to criterion, errors to criterion, and omissions were obtained for each training phase using the above-described criteria. The incorrect responses were divided into three different error types: perseverative, regressive and never-reinforced errors. In the strategy set-shifting phase, these errors were classified as perseverative if the animal responded to the incorrect lever when the visual cue was illuminated above it on more than 12 out of 20 trials (≥60%) within a session. If the animals made <60% incorrect responses, these errors were identified as regressive in all subsequent sessions. Never-reinforced errors occurred when the animal responded on the incorrect lever while the visual cue was presented on the other side. In the reversal learning phase, if the animals made ≥60% incorrect presses (≥24/40 of performed trials), these errors were scored as perseverative. If the animals made <60% incorrect responses, errors were scored as regressive in all subsequent sessions. There were no never-reinforced errors in the reversal learning test.

### Progressive Ratio Test

The PR task was used to evaluate effort-related motivation by quantifying the number of lever presses that a subject was willing to expend to earn a reward in operant conditioning chambers. The experimental paradigm was adapted from the method described previously with minor modifications (Carvalho Poyraz et al., 2016; Tsutsui-Kimura et al., 2017). Briefly, mice were initially trained to press the lever on a fixed ratio (FR)-1 reinforcement schedule whereby a single lever press elicited the delivery of 10 μl of 20% sucrose solution as a reward in the magazine. Only one lever was present, and the allocation of right and left levers was counterbalanced between mice. Following four successive sessions of FR-1 reinforcement schedule, the schedule was upgraded to FR-5 in which five active lever presses triggered the delivery of the reward and lasted for 3 days. Each FR training session lasted 1 h or until the delivery of 60 rewards. After that, all the mice were moved to the PRT. The response ratio schedule during PR testing was calculated according to the formula: [5e (R×0.2)]-5, where R was equal to the number of food rewards already earned plus 1. Thus, the number of responses required to earn a reward followed the order: 1, 2, 4, 6, 9, 12, 15, 20, 25, 32, 40, 50, 62, 77, 95, and so on. The final completed ratio represented the breakpoint. A PR session lasted up to 1 h maximum and failure to press the lever in any 3-min period resulted in the termination of the session.

### Immunofluorescence

Mice were deeply transcardially perfused with 4% paraformaldehyde. Brain slices (30 μm) were sectioned, and immunofluorescence staining was performed on free-floating sections as described previously (Li et al., 2016). Brain slices were incubated with primary anti-A_2A_R (Santa Cruz, 1:50) antibodies overnight. The sections were then rinsed and incubated with Alexa 488 conjugated secondary antibodies (Invitrogen, 1:1,000). The slices were washed and mounted, and images were acquired and quantified as mean integrated optical density using Image Pro Plus software.

### Statistical Analyses

All data were presented as means ± standard error of the mean (SEM). Two-way analysis of variance (ANOVA) for repeated-measures with *post hoc* Bonferroni’s test was used for the comparison of multiple factors (i.e., A_2A_R KD × training sessions). Error subtype and latency to lever were analyzed separately using Two-way ANOVAs, with Treatment as the between-subjects factor and Error Type (perseverative, regressive and never-reinforced errors) or Choice (correct/incorrect) as a within-subjects factor. Significant main effects of Treatment were followed up with multiple comparisons using Bonferroni’s test. Student’s *t*-test was performed for comparison of the two groups (A_2A_R KD vs. control). Statistical comparisons were performed using SPSS statistics version 25. The significance of the differences was considered for *p* < 0.05.

## Results

### Conditional A_2A_R Knockdown in the NAc by the Cre-loxP Strategy

**Figure 1:**
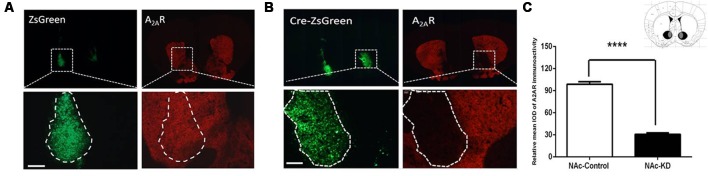
Conditional A_2A_R knockdown (KD) in the nucleus accumbens (NAc) by the Cre-loxP system. **(A,B)** Representative immunofluorescent photomicrographs showing focal KD expression of A_2A_ receptors (A_2A_Rs) in the NAc after injection of AAV8-CAG-ZsGreen **(A)** and AAV8-CAG-Cre-ZsGreen into the A_2A_R^(flox/flox)^ mice **(B)**. The intensity of A_2A_Rs signal (red) was decreased in the overlapping area with Cre-zsGreen expression (**B**; right panels) but not the control (**A**; right panels). **(C)** Schematic illustration of the maximal (black) and minimal (gray) A_2A_R KD areas in the NAc. Quantitative analysis showed that A_2A_Rs expression was markedly reduced in the AAV8-CAG-Cre-zsGreen-transfected regions compared with control virus (*n* ≥ 5). Scale bar = 150 μm. ****p < 0.0001.

To focally knockdown the A_2A_Rs in the NAc, we employed Cre-loxP strategy by injecting AAV8-CAG-Cre-ZsGreen (200 nl) or AAV8-CAG-ZsGreen (control virus) bilaterally into the NAc of A_2A_R^flox/flox^ mice. Three weeks later, the specific areas of virus expression were verified by immunofluorescence. As can be seen in Figure 1C, the black color represents the largest area of virus transfection, and the gray color depicts the smallest one. Furthermore, we observed that the A_2A_Rs expression (the red fluorescence) was reduced selectively in the Cre-expressing regions of the NAc (indicated by green fluorescence, Figure 1B, right panels) but not in the control virus-expressing regions (Figure 1A, right panels). Optical intensity analysis of the A_2A_R immunostaining confirmed that the expression level of A_2A_R in the NAc was decreased by 71% after transfection with AAV8-CAG-Cre-ZsGreen, as compared with the NAc transfected with AAV8-CAG-ZsGreen (Figure 1C). Thus, the A_2A_R expression was selectively and efficiently knocked down in NAc.

### NAc A_2A_R Knockdown Does Not Affect Visual Discrimination but Facilitates Attentional Set-Shifting and Reversal Learning

**Figure 2:**
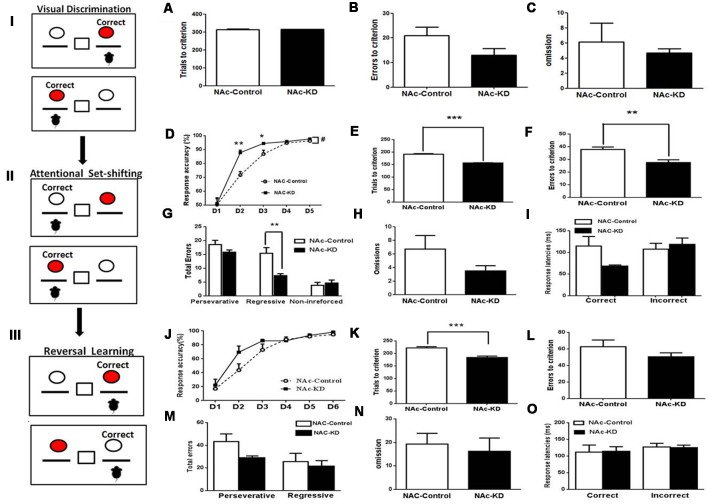
NAc A_2A_R KD did not affect visual discrimination but facilitated attentional set-shifting and reversal learning. (Left panels I, II, III) A schematic illustration of mouse operant cognitive flexibility task. **(A–C)** The NAc A_2A_R blockade did not affect the performance of visual discrimination (total number of trials needed to reach the criterion: *p* > 0.05; total number of errors, *p* = 0.1164; total number of omission, *p* = 0.637, Student’s *t*-test) compared with the control group. **(D–I)** A_2A_R KD in the NAc facilitated attentional set-shifting by decreasing regressive errors. **(D)** A two-way repeated measures analysis of variance (ANOVA) showed that both groups increased their correct response ratio along with increasing sessions (main effect of training session, *F*_(4,44)_ = 124.187, *p* < 0.001), but the correct response ratio in the A_2A_R-KD group increased faster than the control (group effect of group, *F*_(1,11)_ = 10.123, *p* = 0.009). This group effect interacted with the training sessions (group × session interaction, *F*_(4,44)_ = 3.819, *p* = 0.035). **(E,F)** The number of the trials and errors needed to reach the criterion also differed between the two groups (Figure 2E, trials, *p* < 0.05; Figure 2F, errors, *p* < 0.05). There was a significant effect of the NAc A_2A_R KD (Figure 2G, *F*_(1,30)_ = 8.189, *p* < 0.001) and the NAc A_2A_R KD × error type interaction (Figure 2G, *F*_(2,30)_ = 4.565, *p* = 0.0186). Multiple-comparison analysis indicated that NAc A_2A_R KD decreased the regressive errors (*p* < 0.05) but had no effect on the perseverative errors and non-reinforced errors (Figure 2G, both *p* > 0.05). The omissions and correct response latencies showed a decreasing tendency but failed to reach statistical significance after NAc A_2A_R KD (Figure 2H, omissions, *p* = 0.1865; Figure 2I, correct response latencies, *p* = 0.0622). **(J–O)** NAc A_2A_R KD improved reversal learning. **(J)** Response accuracy analysis revealed the session-dependent learning rates across both groups (*F*_(4,44)_ = 99.01, *p* < 0.01), but there was no group difference (*F*_(1,11)_ = 0.679, *p* > 0.05, first 5 days) and group × session interaction effect (*F*_(4,44)_ = 0.812, *p* > 0.05). **(K)** The total number of trials needed for the NAc A_2A_R-KD group was lower than in the control (*p* < 0.001). **(L–O)** NAc A_2A_R KD did not affect the errors to criterion, omissions and response latencies compared to the control group (all* p* > 0.05), but there was a decreasing tendency in the perseverative errors (Figure 2M, *p* = 0.0915). Data are presented as the mean ± standard error of the mean (SEM), ^#^*p* < 0.05, *p < 0.05, **p < 0.01, ***p < 0.001.

Three weeks after the surgery, we implemented the mouse operant cognitive flexibility task to determine the functional involvement of striatal subregion-specific A_2A_Rs in the behavioral cognitive flexibility. This paradigm consists of three different phases: visual discrimination, attentional set-shifting, and reversal learning. In the phase of the visual discrimination, mice were trained to press the specific (left or right) lever above which the cue light was randomly illuminated to get the reward (Figure 2I). NAc A_2A_R KD did not change the total number of trials needed to reach the criterion compared to control (Figures 2A–C, *p* > 0.05). The total number of errors and omissions needed to reach the criterion were also similar between the two groups (Figure 2B, errors, *p* = 0.1164; Figure 2C, omissions, *p* = 0.637). Thus, NAc A_2A_R KD did not affect the performance of visual discrimination.

**Figure 3:**
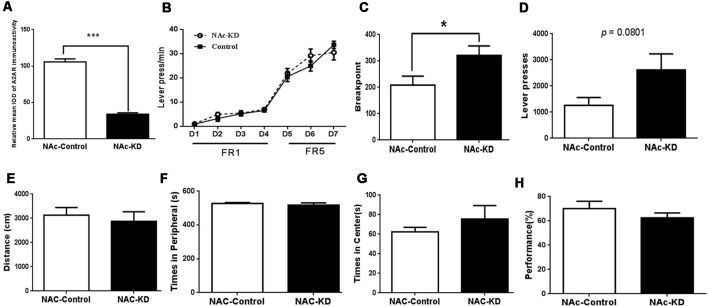
NAc A_2A_R KD-mediated facilitation of cognitive flexibility is not due to the motor activity but associated with enhanced motivation. **(A)** The expression level of NAc A_2A_R in these new groups was validated to have a ~71% decrease after AAV8-CAG-Cre-ZsGreen transfection. **(B)** Both groups of mice gradually increased their lever pressing rates to obtain the reward (*F*_(6,78)_ = 114.564, *p* < 0.01). Two-way ANOVA for repeated-measures revealed neither the main effect of A_2A_R KD (*F*_(1,13)_ = 1.366, *p* = 0.264) nor the manipulation × training session interaction effect (*F*_(6,78)_ = 1.56, *p* = 0.225). **(C)** NAc A_2A_R KD increased the breakpoint (63.2% increase, *p* = 0.0477) and **(D)** had a decreased tendency in the total number of presses (121.4% increase, *p* = 0.0801). **(E)** NAc A_2A_R KD did not affect the total moving distance in the open-field test in comparison with the control (*p* > 0.05, Student’s *t*-test). **(F,G)** NAc A_2A_R KD did not alter the time spent in the peripheral (**F**, *p* > 0.05) vs. central areas (**G**, *p* > 0.05). **(H)** NAc A_2A_R KD also did not affect the performance of spontaneous alternations in Y-maze (**H**, *p* > 0.05). Data are presented as the mean ± SEM, *p < 0.05, ***p < 0.001.

After reaching the criterion of an average correct response of >75% on three consecutive days in the visual discrimination test, the mice were moved to the attentional set-shifting phase which required shifting attention away from a visual cue-reinforced dimension to the spatial location-reinforced dimension to obtain the reward. In this phase, the animals were required to press the non-preferred lever to obtain the reward regardless of the position of the cue light (Figure 2II). Both control and NAc A_2A_R KD groups increased their correct response ratio with increasing sessions (Figure 2D, the training session effect, *F*_(4,44)_ = 124.187, *p* < 0.001, two-way ANOVA for repeated measures). However, the correct response ratio in the A_2A_R KD group increased much faster than in the control (Figure 2D, the group effect, *F*_(1,11)_ = 10.123, *p* = 0.009) and this effect was dependent on the training sessions (Figure 2D, the group × session interaction: *F*_(4,44)_ = 3.819, *p* = 0.035). There were group differences in the second and third training sessions (Figure 3D, both *p* < 0.05). The number of the trials and errors needed to reach the criterion also differed between the two groups (Figure 2E, trials, *p* < 0.05; Figure 2F, errors, *p* < 0.05). Furthermore, error type analysis revealed a significant effect of the NAc A_2A_R KD (Figure 2G, *F*_(1,30)_ = 8.189, *p* < 0.001) and the NAc A_2A_R KD × error type interaction (Figure 2G, *F*_(2,30)_ = 4.565, *p* = 0.0186). Multiple-comparison analysis indicated that NAc A_2A_R KD significantly decreased the regressive errors (*p* < 0.05) but had no effect on perseverative errors and non-reinforced errors (Figure 2G, both *p* > 0.05). The omissions and correct response latencies showed a decreasing tendency but failed to reach statistical significance after NAc A_2A_R KD (Figure 2H, omissions, *p* = 0.1865; Figure 2I, correct response latencies, *p* = 0.0622). Thus, NAc A_2A_R KD did not affect the performance of visual discrimination but facilitated attentional set-shifting by decreasing regressive errors.

Following the set-shifting phase, reversal learning was implemented during which the reward contingencies were reversed (i.e., the reinforced lever was opposite to the lever in the set-shifting phase, Figure 2III) until the criterion was achieved. Response accuracy analysis revealed session-dependent learning rates across both groups (Figure 2J, *F*_(4,44)_ = 99.01, *p* < 0.01). However, two-way ANOVA revealed that there was no group difference (*F*_(1,11)_ = 0.679, *p* > 0.05) and group × session interaction effect (*F*_(4,44)_ = 0.812, *p* > 0.05). Nonetheless, NAc A_2A_R KD was associated with an improved tendency in the correct response accuracy on Day 2 compared to the control. Moreover, NAc A_2A_R KD facilitated the mice to reach the criterion earlier with the total number of trials needed to reach the criterion being lower than that in the control (Figure 2K, *p* < 0.001). However, in addition to having a decreasing tendency in the perseverative errors (Figure 2M, *p* = 0.0915), the errors to criterion, the omissions, and the response latencies were indistinguishable between the two groups (Figures 2L–O, *p* > 0.05).

### NAc A_2A_Rs Knockdown-Mediated Facilitation of Cognitive Flexibility Is Not Attributed to Motor Activity but Associated With Enhanced Motivation

The decreasing tendency of the omission number and the correct response latency induced by NAc A_2A_R KD in the attentional set-shifting test prompted us to evaluate the effect of NAc A_2A_R KD on the effort-related motivation by the PRT using a separate set of NAc A_2A_R KD and control mice. The selective KD of NAc A_2A_R (71%) by transfection with the AAV8-CAG-Cre-ZsGreen virus in these new groups was confirmed by fluorescence histochemistry (Figure 3A). In the training stage, both groups of mice gradually increased their lever pressing rates to obtain the reward (Figure 3B, *F*_(6,78)_ = 114.564, *p* < 0.01). We did not observe the main effect of NAc A_2A_R KD (Figure 3B, *F*_(1,13)_ = 1.366, *p* = 0.264) nor NAc A_2A_R × training course interaction (*F*_(6,78)_ = 1.56, *p* = 0.225, two-way ANOVA for repeated measures). However, NAc A_2A_R KD increased the breakpoint (Figure 3C, 63% increase, *p* = 0.0477) and had an increasing tendency in the total number of presses (Figure 3D, 121% increase, *p* = 0.0801) in the test stage.

To exclude the possible confounding effect of NAc A_2A_Rs KD on locomotion, anxiety-like behavior, and working memory, we evaluated the locomotor activity in the open-field test and spontaneous alternations in the Y-maze test. NAc A_2A_R KD did not affect locomotion (Figure 3E, *p* > 0.05, Student’s *t*-test) and the residence time in both central (Figure 3G, *p* < 0.05) and peripheral (Figure 3F, *p* < 0.05,) areas were indistinguishable between the control and NAc A_2A_R KD groups. There was also no difference in possible working memory performance by the spontaneous alternation in the Y-maze test between these two groups (Figure 3H, *p* < 0.05).

### Conditional A_2A_R Knockdown in the DMS by the Cre-loxP Strategy

**Figure 4:**
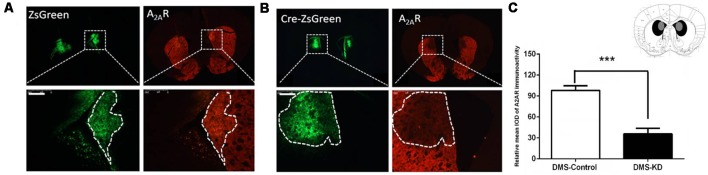
Conditional A_2A_R KD in the dorsomedial striatum (DMS) *via* the Cre-loxP system. **(A,B)** We employed the same Cre-loxP strategy to selectively knockdown the A_2A_R in the DMS by injecting AAV8-CAG-ZsGreen (control virus) or AAV8-CAG-Cre-ZsGreen bilaterally into the DMS of A_2A_R^flox/flox^ mice. A_2A_R expression (the red fluorescence) was reduced selectively in the Cre-expressing regions of the DMS (**B**; right panels) but not in the control DMS (**A**; right panels). **(C)** Schematic illustration of the maximal (black) and minimal (gray) A_2A_R KD areas in the DMS and optical intensity analysis confirmed that the expression level of A_2A_R in the DMS was decreased by 74% compared to the control group. ***p < 0.001.

Due to the heterogeneity of the striatum, we further examined the contributions of the DMS A_2A_Rs to cognitive flexibility. To selectively knockdown the A_2A_Rs in the DMS, the same Cre-loxP strategy was used by injecting AAV8-CAG-Cre-ZsGreen or AAV8-CAG-ZsGreen (control virus) bilaterally into the DMS of A_2A_R^flox/flox^ mice. Three weeks later, the specific area of virus expression was verified by immunofluorescence, as can be seen in the Figure 4C in which the black color represents the largest area of virus transfection and the gray color depicts the smallest one. Furthermore, we observed that A_2A_Rs expression (the red fluorescence) was reduced selectively in the Cre-expressing regions of the DMS (indicated by green fluorescence, Figure 4B, right panels; but not in the control DMS, Figure 4A, right panel). Optical intensity analysis of the A_2A_R immunohistochemistry (Figure 4C) confirmed that the expression level of A_2A_Rs in the DMS was decreased by 74%, compared with the control groups.

### DMS A_2A_R Knockdown Does Not Affect Visual Discrimination, Attentional Set-Shifting and Reversal Learning

**Figure 5:**
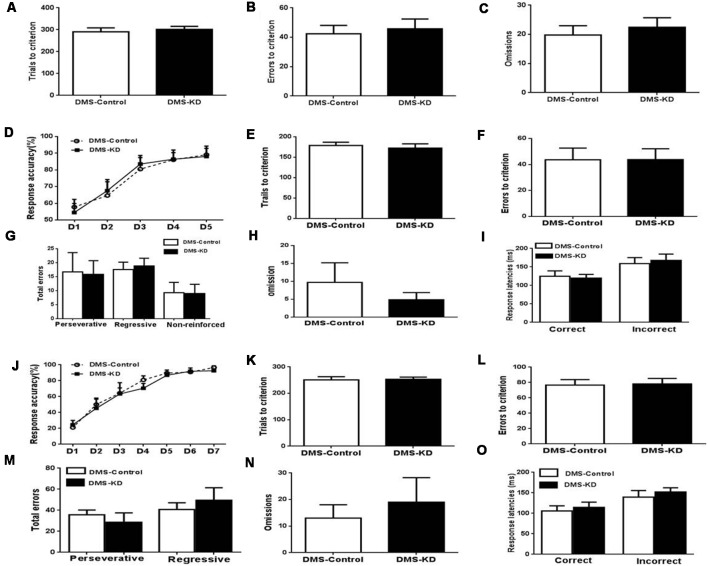
DMS A_2A_R KD did not affect visual discrimination, attentional set-shifting, and reversal learning. **(A–C)** A_2A_R KD in the DMS did not affect the performance in task acquisition of visual discrimination (all* p* > 0.05). **(D–I)** A_2A_R KD in the DMS did not affect the attentional set-shifting. **(D)** A two-way repeated measures ANOVA showed the main effect of the training session (*F*_(4,48)_ = 54.609, *p* < 0.01), but no effect of the manipulation (*F*_(1,12)_ = 0.17, *p* = 0.9) and training × manipulation interaction (*F*_(4,48)_ = 0.412, *p* = 0.799). **(E–I)** There was no significant difference in the trial number (*p* = 0.6328), learning errors (*p* = 0.991), omission (*p* = 0.4199) and response latencies (*p* > 0.05). **(J–O)** Knockdown of A_2A_Rs in the DMS did not affect reversal learning. **(J)** Response accuracy analysis revealed the session-dependent learning rates across DMS A_2A_R KD and control groups (*F*_(4,32)_ = 37.371, *p* < 0.01, two-way ANOVA with repeated measures), but no group difference (*F*_(1,8)_ = 0.175, *p* > 0.05, first 5 days) and group × session interaction effect (*F*_(4,32)_ = 0.382, *p* > 0.05). **(K–O)** DMS A_2A_R KD did not affect total trials, errors to criterion, omissions, and response latencies, as compared to the control group (all* p* > 0.05).

Similarly, the DMS A_2A_R KD and control mice were tested by mouse operant cognitive flexibility task to decipher the possible heterogeneous function of striatal subregion A_2A_Rs. In the visual discrimination stage, DMS A_2A_R KD also did not affect the performance of visual discrimination (Figures 5A–C, *p* > 0.05, Student’s *t*-test). Two-way ANOVA analysis revealed that there was a main effect of the training session (Figure 5D, *F*_(4,48)_ = 54.609, *p* < 0.01), but in contrast to NAc A_2A_R KD, neither the effect of the DMS A_2A_R KD (*F*_(1,12)_ = 0.17, *p* = 0.9) nor the training × DMS A_2A_R KD interaction (*F*_(4,48)_ = 0.412, *p* = 0.799) were observed in the attentional set-shifting phase. Also, the trial number (*p* = 0.6328), learning errors (*p* = 0.991), omission (*p* = 0.4199) and response latencies (*p* > 0.05) were all indistinguishable between the DMS A_2A_R KD and control groups (Figures 5E–I). Similarly, we found that DMS A_2A_R KD did not affect the performance (i.e., the number of correct responses, errors, omissions and response latencies) in the reversal phase (Figures 5J–O). Collectively, these data suggested that DMS A_2A_R KD did not affect the performance in visual discrimination, attentional set-shifting, and reversal learning.

### DMS A_2A_R Knockdown Does Not Affect Locomotion but Enhances Motivation

**Figure 6:**
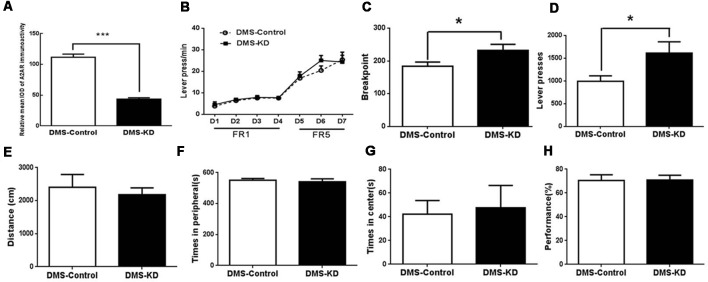
DMS A_2A_R KD did not affect locomotion but enhanced motivation. **(A)** The expression level of DMS A_2A_R in these new groups was decreased by ~70% after AAV8-CAG-Cre-ZsGreen transfection. **(B–D)** DMS A_2A_R KD enhanced motivation. **(B)** There was no significant difference in the training stage between these two groups (effect of training session, *F*_(6,96)_ = 51.558, *p* < 0.05; DMS A_2A_R KD × training course interaction effect, *F*_(6,96)_ = 1.57, *p* > 0.05; effect of DMS A_2A_R KD, *F*_(1,16)_ = 0.6, *p* > 0.05, two-way ANOVA with repeated measures). **(C,D)** DMS A_2A_R KD increased the breakpoint by 30.6% (*p* = 0.0233, Student’s *t*-test) and a total number of presses by 62.6% (*p* = 0.0179, Student’s *t*-test) in the PRT stage. **(E–H)** DMS A_2A_R KD did not affect locomotion, the residence time in the central and peripheral area in the open-field test, and working memory by a spontaneous alternation in Y-maze, compared to the control (all *p* > 0.05). *p < 0.05; ***p < 0.001.

Using a new set of the mice with confirmed KD (by 70%) of DMS A_2A_R after transfection with AAV8-CAG-Cre-ZsGreen (Figure 6A), we showed that DMS A_2A_R KD also did not affect locomotion, the residence time in the central and peripheral area in the open-field test and possible working memory by a spontaneous alternation in Y-maze compared to the control (Figures 6E–H, all *p* < 0.05). However, PR task revealed that the breakpoint (Figure 6C, 30.6% increase, *p* = 0.0233) and total number of presses (Figure 6D, 62.6% increase, *p* = 0.0179) were also significantly increased by DMS A_2A_R KD, although there were no significant differences in the training stage between these two groups (Figure 6B, effect of training session, *F*_(6,96)_ = 51.558, *p* < 0.05; DMS A_2A_R KD × training course interaction effect, *F*_(6,96)_ = 1.57, *p* > 0.05; effect of DMS A_2A_R KD, *F*_(1,16)_ = 0.6, *p* > 0.05). Thus, DMS A_2A_R KD can enhance motivation.

## Discussion

### The Striatopallidal A_2A_Rs in the NAc Modulate Cognitive Flexibility by Facilitating Strategy Shifting

The important finding in this study is that NAc A_2A_R KD enhances cognitive flexibility by increasing set-shifting as well as reversal learning. First, NAc A_2A_Rs blockade improved attentional set-shifting as evident from the increased response accuracy and decreased the number of trials to reach the criterion. The enhanced cognitive flexibility by NAc A_2A_R KD is associated with the decreased regressive errors, indicating that the A_2A_R KD mice can efficiently identify the newly reinforced choice and strongly maintain this new response, or accelerate the learning about the irrelevant stimuli (the right or left lever was irrelevant in the visual discrimination but turned into the relevant stimuli in the stage of the attentional set-shifting). This finding is consistent with the other reports showing that inactivation of the NAc enhanced learning about the irrelevant stimuli in the set-shifting (Tai et al., 1995; Jongen-Rêlo et al., 2002; Floresco et al., 2006). Thus, NAc A_2A_R KD selectively improved attentional set-shifting by increasing the ability of learning and maintaining the new extradimensional strategy.

Furthermore, NAc A_2A_R KD also improves reversal learning as evident from the decreased number of trials to reach the criterion and the increased correct response accuracy on day 2 in the phase of reversal learning. This improvement is associated with apparently decreasing perseverative errors, indicating the increased inhibition of previously learned (old) strategy to facilitate the strategy shifting. This finding is consistent with our previous finding that A_2A_R KO increases performance in the omission test, in which the mice learn to suppress lever pressing for 20 s in order to obtain the reward, indicating an increased behavioral inhibition (Yu et al., 2009).

The facilitation of search for a new strategy in the set-shifting phase by reduced regressing errors and inhibition of the previous strategy in the reversal phase by the tendency for a reduction in perseverative errors suggests that NAc A_2A_R controls cognitive flexibility by two distinct processes that are distinctly controlled by glutamatergic inputs from mPFC and orbitofrontal cortex (OFC) into the NAc, respectively (Birrell and Brown, 2000; McAlonan and Brown, 2003; Cui et al., 2018). Importantly, modulation of both processes leads to the enhanced strategy shifting by the A_2A_R KD in the NAc. This view is also consistent with that A_2A_R KO enhances goal-directed behavior in instrumental conditioning (Yu et al., 2009) and strategy shifting in water maze paradigm (Wei et al., 2011), and is supported by the finding that caffeine (a non-specific antagonist of A_2A_R) treatment significantly improves attention and cognitive deficits in an attentional deficit and hyperactivity disorder (ADHD) animal model (Pandolfo et al., 2013). Furthermore, the NAc A_2A_R KD seems only to modulate the early phases of set-shifting instead of consolidating the new searching strategy, since the response of reversal learning on the Day 1 was similar between these two groups and the NAc A_2A_R KD even improves reversal learning by decreasing the number of trials to reach the criterion. These indicate that NAc A_2A_R may only control short-term memory or goal-directed behavior, which is also supported by our unpublished data which reveal that NAc A_2A_R KD can improve short-term working memory in a delayed non-match-to-place (DNMTP) task and goal-directed behavior in the instrumental behavior.

Collectively, these findings suggest that NAc A_2A_R enhances cognitive flexibility by facilitating strategy shifting *via* increasing the ability of learning and maintenance of new extradimensional strategy and possible inhibition of intradimensional old strategy. Except for the D1R agonists (Haluk and Floresco, 2009), most studies with pharmacological and genetic manipulation of neuromodulators and focal lesioning produced almost exclusive impairment of cognitive flexibility (Ding et al., 2014; Parikh et al., 2016b; Grospe et al., 2018). Our finding may shed new light on the striatopallidal pathway control of cognitive flexibility as the A_2A_R is selectively expressed in the striatopallidal neurons and the A_2A_R KD is expected to reduce the striatopallidal neuron activity. The previous studies have produced different results on cognitive flexibility. For example, optogenetic (ChR2) activation in DMS (Wang et al., 2019) or by toxin-induced depletion of the striatopallidal pathway in NAc (Yawata et al., 2012) can facilitate or impair cognitive flexibility, while optogenetic (NpHR) silencing of striatopallidal pathway in DMS produces no effect (Wang et al., 2019). In this regard, NAc A_2A_R KD selectively improving cognitive flexibility, combined with the noted safety profile of A_2A_R antagonists and caffeine in clinical phase III trials for motor benefits in Parkinson’s disease (Chase et al., 2003) suggests that pharmacological targeting striatal A_2A_R may represent a novel treatment strategy for the deficits of cognitive flexibility in various neuropsychiatric disorders.

### The NAc A_2A_R KD May Enhance Cognitive Flexibility by Modulating Motivation/Attention

The mechanism underlying the NAc A_2A_R KD-mediated facilitation of cognitive flexibility is not clear. Notably, this facilitation by NAc A_2A_R KD is not attributed to the mnemonic process or possible working memory, as NAc A_2A_R KD did not affect the performance in the visual discrimination phase (Figures 2A–C) and the acquisition of lever pressing in the PRT (Figure 3A) and spontaneous alternations in Y-maze. This is consistent with previous finding that this task is specifically sensitive to the manipulation to disruption of cognitive flexibility (i.e., set-shifting and reversal learning) but relatively insensitive to mnemonic process since manipulation of glutamate and dopamine signaling mainly alter cognitive flexibility without affecting visual discrimination (Parikh et al., 2016b; Cui et al., 2018; Kato et al., 2018). Moreover, this NAc A_2A_Rs-mediated modification of cognitive flexibility is neither confounded by motor activity nor possibly anxiety-like behavior (Figures 3E–G). As the motivational factor is critical to the control of cognitive flexibility (Liu and Wang, 2014) and NAc is the critical locus for motivational control, we propose that NAc A_2A_Rs may improve cognitive flexibility with facilitated strategy shifting by enhancing the motivation. This proposal is supported by the finding of a decreased tendency of the number of omissions and correct response latency induced by NAc A_2A_R KD in attentional set-shifting test (Figures 2H–I). This contention is further validated by the finding that NAc A_2A_R KD increases the breakpoint and enhances the motivation in PRT. The enhanced response induced by A_2A_R KD in PRT can be explained by an enhanced sensitivity to reinforcement, rapid initiation of lever pressing or enhanced persistence of the action. These results are in line with the findings that A_2A_R antagonism and genetic deletion can improve effort-based decision making (Pardo et al., 2012; López-Cruz et al., 2018). We noted that A_2A_R KD in both NAc and DMS increased motivation (i.e., breakpoint in PRT), consistent with the previous study that showed the inhibition of the indirect pathway in either the NAc or DMS leads to enhanced motivation (Carvalho Poyraz et al., 2016). However, the NAc A_2A_R KD produces a higher breakpoint than DMS A_2A_R KD. It is possible that different intensities of motivation may lead to the different control of cognitive flexibility by NAc vs. DMS A_2A_R KD and additional studies are needed to clarify this issue.

Additionally, the reduced response latency in the set-shifting after NAc A_2A_R KD may indicate an increased attention. The enhanced response induced by A_2A_R KD in the set-shifting can also be attributed to an enhanced sensitivity to environmental sensory stimulation (or enhanced attention to the detection of novel features). The NAc receives dense direct glutamatergic projections from vHIP with sensory inputs to generate new attention (Voorn et al., 2004; Mannella et al., 2013; Floresco, 2015), from the paraventricular thalamus to track context-dependent salience (Zhu et al., 2018), from the BLA to modulate cue-triggered motivated behavior (Stuber et al., 2011) and dopaminergic projections from VTA to control attention, approach initiation and flexible reward-seeking (Syed et al., 2016; Boekhoudt et al., 2018; Cui et al., 2018). These pathways confer the NAc the unique feature to modulate cognitive flexibility by modifying sensitivity to environmental sensory stimulation and attention to the detection of novel features, resulting in action selection with motivation.

Dopamine signaling in the striatum is also critical to cognitive flexibility control (Parikh et al., 2016b; Cui et al., 2018). Individual differences in the dopamine D2-type receptor (D2R) levels in the caudate nucleus of human subjects and monkeys correlate with performance in a discrimination reversal task (Horst et al., 2019). As striatal A_2A_Rs exert an inhibitory effect on D2R signaling, possibly through the A_2A_R-D2R heterodimers in the striatopallidal neurons where they almost exclusively colocalized, it can be speculated that NAc A_2A_R KD may facilitate cognitive flexibility with enhanced motivation by modulating D2R signaling in the striatum. Consistent with this view, global D2R knockout has been shown to disrupt reversal learning in mice (Horst et al., 2019). However, focal injection of the D2R agonist quinpirole into NAc produces opposite effects to NAc A_2A_R KD, i.e., disruption of both set-shifting and reversal learning (Haluk and Floresco, 2009; Horst et al., 2019). Whether NAc A_2A_Rs control cognitive flexibility by interacting with the D2R remains to be clarified. In addition, A_2A_R activity can also modulate glutamate signaling through the antagonistic interactions with NMDARs and metabotropic glutamate receptor 5 (mGlu5) in the striatum (Parsons et al., 2007; Zhu et al., 2016, 2018). In agreement with this proposal, NMDAR antagonists have been shown to impair both set-shifting and reversal learning (Darrah et al., 2008; Ding et al., 2014).

### Striatopallidal A_2A_Rs Exert NAc- and DMS-Specific Control of Cognitive Flexibility

Another noted observation is that striatopallidal A_2A_Rs exert NAc- and DMS-specific control of cognitive flexibility. Consistent with the critical role of the NAc in modulating the cognitive flexibility (Haluk and Floresco, 2009; Ding et al., 2014; Cui et al., 2018), NAc A_2A_R KD enhances cognitive flexibility (i.e., both set-shifting and reversal learning) by facilitating strategy shifting. By contrast, DMS A_2A_R KD is devoid of effects on cognitive flexibility, as evident by the lack of effects of DMS A_2A_R KD on set-shifting and reversal learning. As DMS has been shown to control cognitive flexibility (Aoki et al., 2015; Grospe et al., 2018), the lack of effects of DMS A_2A_R on cognitive flexibility indicates that neuromodulators other than the A_2A_R (such as acetylcholine and glutamate signaling) may mediate DMS control of cognitive flexibility. Furthermore, DMS A_2A_R KD can enhance goal-directed behavior (Li et al., 2016) and working memory (Li et al., 2018), two behavioral elements involved in cognitive flexibility control. Thus, other behavioral elements such as attention and impulsivity control (associated mainly with the NAc function) may play critical roles in this control. The dissociable function of striatal subregion A_2A_Rs in modulating cognitive flexibility collaborate with several previous studies including ours showing that the cortico-striatal A_2A_Rs can exert different or opposite effects on behaviors. For example, we have recently shown that prefrontal and striatal A_2A_Rs have the opposite effect on working memory (Li et al., 2018), fear memory (Wei et al., 2014) and psychomotor activity (Shen et al., 2008). Similarly, selective down-regulation of A_2A_Rs in the prefrontal cortex has been shown to cause an impulsive-like behavior in the delay-based cost-benefit decision-making paradigm (Leffa et al., 2018), while pharmacological blockade or genetic inactivation of A_2A_Rs can reverse the impairment induced by D2R antagonist in an effort-related cost-benefit decision-making paradigm (Pardo et al., 2012). Also, the distinct functions of the cortico-striatal A_2A_Rs at the presynaptic vs. postsynaptic sites may underlie opposite control of cognitive behaviors by A_2A_Rs in the prefrontal cortex and striatum, as different behaviors may be preferentially controlled by postsynaptic striatal A_2A_Rs (such as working memory; Li et al., 2018) or presynaptic cortical A_2A_Rs (such as THC self-administration; Tebano et al., 2004; Justinová et al., 2014). This opposite control of behaviors by A_2A_Rs in different brain regions (at the presynaptic vs. postsynaptic levels) may confer A_2A_Rs with the ability to keep each behavior in balance and to fine-tune behaviors.

As the A_2A_R signaling and functional interaction with other neurotransmitters are similar in different subregions of the striatum (Svenningsson et al., 1999), the functional divergence of striatal subregion-specific A_2A_Rs in controlling strategy shifting may be primarily attributed to the distinct input-output mapping. NAc mainly receives inputs from mPFC, orbital prefrontal cortex, vHIP, BLA and VTA and projects to ventral pallidum whereas DMS mainly receives inputs from mPFC, intralaminar thalamic nuclei and SNc and projects to globus pallidus external (Britt et al., 2012; Papp et al., 2012; Hunnicutt et al., 2016; Kato et al., 2018). Functionally, the hippocampus is essential in processing the relationships between different stimuli and recognition of novelty (Mannella et al., 2013) and vHIP-NAc stimulation may increase VTA dopamine neuron population activity (Floresco et al., 2001). The BLA also plays a crucial role in forming associations between neutral stimuli and guiding action selection in situations involving reward uncertainty (McLaughlin and Floresco, 2007; Bercovici et al., 2018). Also, the VTA dopamine preferentially projecting to NAc is critical to the control of motivation, rewarding behavior, and affection, whereas the SNc dopamine mostly projecting to DMS mainly contributes to the motor and possibly motivation functions (Le Moal and Simon, 1991; Nieoullon and Coquerel, 2003; Björklund and Dunnett, 2007). Moreover, activation of VTA dopaminergic neurons impaired sustained attention (Boekhoudt et al., 2017) and increased responsiveness to sucrose and enhanced motivation for the reward in the PRT (Boekhoudt et al., 2018) while activation of SNc dopaminergic neurons impaired attention and delayed responsiveness and had no effects on sucrose seeking and motivation (Boekhoudt et al., 2017, 2018); meanwhile, a recent study has demonstrated that NAc and dorsal striatum have differences in the sensitivity and timing of D2-receptor signaling with higher sensitivity for dopamine in the NAc by preferential coupling to G_αo_ (Engeln et al., 2018; Marcott et al., 2018). The subregional heterogeneities of D2R signaling and function in the striatum may also underlie distinct control of cognitive flexibility by NAc vs. DMS A_2A_Rs.

It should be noted that NAc A_2A_Rs may control cognitive flexibility by striatal collateral control and striatal local microcircuits involving interneurons and glial cells, as recent studies have demonstrated that there are collateral synapses between striatopallidal neurons and striatonigral neurons (Lalchandani et al., 2013; Wei et al., 2017) and the striatonigral neurons (so-called direct pathway) played important roles in controlling cognitive flexibility (Haluk and Floresco, 2009; Wang et al., 2019). Meanwhile, the striatal cholinergic interneurons and astrocyte calcium signaling also can modulate set-shifting and repetitive behavior possibly by controlling local microcircuits (Aoki et al., 2015; Yu et al., 2018). However, to confirm these possible mechanisms, additional studies are needed to dissect out the circuit and neurochemical basis of the differential control of cognitive flexibility by NAc vs. DMS A_2A_Rs.

## Data Availability

The raw data supporting the conclusions of this manuscript will be made available by the authors, without undue reservation, to any qualified researcher.

## Ethics Statement

The animal protocols were approved by the Institutional Ethics Committee for Animal Use in Research and Education at Wenzhou Medical University, China.

## Author Contributions

J-FC and JZ conceived and designed the experiments and wrote the article. JZ, BW, XL, YD, TL, and XC performed the experiments. JZ, WZ, GW, and SV analyzed the data. JZ, WZ, WG, and XC contributed reagents, materials and analysis tools. SV also contributed to the manuscript text edition.

## Conflict of Interest Statement

The authors declare that the research was conducted in the absence of any commercial or financial relationships that could be construed as a potential conflict of interest.
